# A new hexapeptide from the leader peptide of rMnSOD enters cells through the oestrogen receptor to deliver therapeutic molecules

**DOI:** 10.1038/srep18691

**Published:** 2016-01-04

**Authors:** Antonella Borrelli, Antonietta Schiattarella, Roberto Mancini, Alessandra Pica, Maria Laura Pollio, Maria Grazia Ruggiero, Patrizia Bonelli, Viviana De Luca, Franca Maria Tuccillo, Clemente Capasso, Enrico Gori, Marina Sanseverino, Andrea Carpentieri, Leila Birolo, Piero Pucci, Jean Rommelaere, Aldo Mancini

**Affiliations:** 1 Molecular Biology and Viral Oncology, National Cancer Institute “Fondazione Pascale”, Naples, Italy; 2 Department of Biology, University of Naples, Federico II, Italy; 3 Institute of Protein Biochemistry, C.N.R., Naples, Italy; 4 Department of Statistics, University of Udine, Udine, Italy; 5 INBIOS - Institute of Genetic and Biophysics, C.N.R., Naples, Italy; 6 Department of Biotechnology, Friedrich-Loeffler-Institut, Neustadt, Germany; 7 Department of Chemical Sciences, University of Naples, Federico II, Italy; 8 Deutsches Krebsforschungszentrum, Infection and Cancer Program, Abt.F010, Heidelberg, Germany; 9 Laedhexa Biotechnologies Inc., QB3@953, San Francisco, CA, USA

## Abstract

A 24-amino acid leader peptide of a new human recombinant manganese superoxide dismutase can enter cells and carry molecules. Here, we demonstrated that six of the 24 amino acids penetrate cells through a particular gate represented by a specific amino acid sequence of the oestrogen receptor (ER). We analysed the internalization of the synthetic hexapeptide and the cytotoxic activity of the hexapeptide conjugated to cisplatin on a cell line panel. In most cell lines, the hexapeptide delivered an amount of cisplatin that was 2 to 8 times greater than that released by cisplatin when the drug was used alone. This increased delivery increases the therapeutic index of cisplatin and reduces side effects caused by a high dosage or long-term treatment times. We may consider this hexapeptide a new molecular carrier to deliver molecules with therapeutic activity into ER^+^ cells for diagnostic purposes and clinical or immune therapy.

Superoxide dismutases (SODs) are antioxidant enzymes that catalyse the O_2_^–^ free radical dismutation of hydrogen peroxide (H_2_O_2_), thereby preventing the accumulation of these activated oxygen species. H_2_O_2_ can be further converted into H_2_O and molecular oxygen (O_2_) by catalase and glutathione peroxidase. At least 3 types of SODs are present in human tissues^1^, including cytoplasmic Cu/Zn-SOD, extracellular Cu/Zn-SOD (ecSOD)^2^ and mitochondrial manganese (Mn) SOD (MnSOD). The manganese-dependent MnSOD-2 is characteristic of aerobic organisms and is composed of four homologous 24-kDa subunits^3^. MnSOD is synthesized in the cytoplasm and then driven into the mitochondrial matrix via its leader sequence, consisting of 24 amino acids (aa). This peptide is subsequently cleaved, resulting in a mature and enzymatically active protein that plays a pivotal role within the cell. While MnSOD has been reported to protect cells from various types of insults and suppress apoptosis^4^, the compound may also be deleterious and impede cell proliferation under certain circumstances^5^,^6^. Thus, SODs appear to control multiple reactions essential to the determination of cell fate, particularly for cancer cells^7^,^8^. The excess production of reactive oxygen species (ROS) leads to cell damage, ageing and a large number of diseases; however, none of the commercially available SODs are administrable and able to enter cells. Moreover, these SODs are inactivated or excreted by the kidney^9^.

Recently, a new isoform of human MnSOD was isolated and obtained in a synthetic recombinant form and termed rMnSOD. This isoform is different due to its ability to enter cells, its intense antioxidant and antitumour activities and its easy administration by injection^10^,^11^,^12^. rMnSOD appears to be very effective at O_2_^**–**^scavenging both intra- and extracellularly and at improving pathological conditions associated with increased oxidative stress^13^. In addition, rMnSOD shows a good biodistribution *in vivo,* particularly in the liver^14^, suggesting that it is well suited for correcting hepatic oxidative stress. Moreover, rMnSOD is radioprotective for healthy cells and radiosensitive for cancer cells^15^, and it displays a specific and selective cytotoxic activity against tumour cells expressing the oestrogen receptor (ER)^16^. rMnSOD also provides protection to rat kidneys treated with cyclosporine-A, allowing for the recovery of 80% of their glomerular filtrate^17^. Previously, we showed that rMnSOD enters cells by means of its 24-aa leader peptide, which represents the rMnSOD molecular carrier^18^.

This feature of the 24-aa leader peptide that it can enter cells expressing the ER while bound to different molecules encouraged us to investigate this phenomenon. We crosslinked the 24-aa leader peptide with the ER and performed a mass spectrometric analysis. We identified the aa sequence of the leader peptide linked to the ER. The result of this assay was the identification of a 6-aa sequence that participates in ER binding. We concluded that this 6-aa sequence is a molecular carrier, allowing rMnSOD to enter cells. The present study examined how this hexapeptide was able to enter cells expressing ER and deliver into the cells the material bound to it.

## Results

### Identification of the rMnSOD peptide involved in the interaction with ER

Identification of the minimal rMnSOD peptide recognized by the ER was pursued by chemical crosslinking experiments followed by mass spectrometric analyses (details in the supplementary document, Mass Spectrometry Data). N-ε-maleimidocaproyl- oxysulfosuccinimide ester (Sulfo-EMCS), a hetero-bifunctional reagent, was selected as a crosslinker to take advantage of the Cys residue occurring within the 24-aa rMnSOD leader peptide. This reagent can form a covalent bond between Cys and Lys residues juxtaposed at an appropriate distance. The 24-residue peptide was then incubated with the ER protein, and the crosslinking reaction was performed in parallel with a control experiment where the reagent was omitted. Following chemical modification, both the sample and control were enzymatically doubly digested with V8 protease and trypsin, and the resulting peptide mixture was directly analysed by mass spectrometry matrix-assisted laser desorption/ionization (MALDI-TOF). The mass signals recorded in the spectrum were assigned to the corresponding peptides within the anticipated ER sequence on the basis of their mass value and the proteases specificity. The mass mapping profiles of both the crosslinked sample and the untreated ER protein were compared. The greatest differences in the two profiles were clearly identified in the 450–470 region of the ER sequence; the mass signals mapping to this region could only be detected in the untreated ER protein, whereas they were absent in the crosslinked sample. Moreover, the chemically modified ER mass profile showed the occurrence of two signals at m/z 3300.7 Da and 31714.4 Da that were absent in the control spectra. These signals were respectively interpreted as arising from the rMnSOD hexapeptide ***AVCGTG***covalently linked to fragments 450–471 and 450–470 of the ER protein ***SIILLNSGVYTFLSSTLKSLEE***through the Sulfo-EMCS reagent. These results demonstrated that the small hexapeptide AVCGTG from the rMnSOD, similarly to the 24-residue fragment described above, could be recognized by the ER protein. This suggests that the hexapeptide plays the same biological role as the larger 24 aa peptide.

### Synthesis of a hexapeptide (*AVCGTG)* and a scrambled hexapeptide (*GAVCGT*) conjugated to fluorescein

Fluorescein 6-residue peptides were synthesized by Fmoc-solid phase peptide synthesis (SPPS) conditions using 9-fluorenylmethoxycarbonyl (Fmoc)-chemistry. The products were released from the solid support and freed from protecting groups by acidic cleavage, precipitated with ether, filtered, re- suspended in water and lyophilized. Purification of the crude products by reversed-phase high- performance liquid chromatography (RP-HPLC) gave the desired products with 90–95% purity and the correct molecular weight (MW), as confirmed by mass spectrometry (MALDI-TOF).

### Synthesis of the hexapeptide (AVCGTG) conjugated to cisplatin

A platinum (Pt) (II)-chelating 6-residue peptide was synthesized by solid phase synthesis using Fmoc-chemistry. The chelant moiety and the coordination of Pt (II) through PtCl_2_ were performed according to the literature^19^. The product was released from a solid support and freed from protecting groups by acidic cleavage; it was then precipitated with ether, filtered, re- suspended in water and lyophilized. Purification of the crude product by RP-HPLC gave the desired product with 90–95% purity and the correct MW as confirmed by mass spectrometry (MALDI-TOF).

### Uptake of Pt exerted by the hexapeptide conjugated to cisplatin assessed by atomic absorbance spectrophotometric analysis

The presence of Pt in cancer cells following the treatment of target cells (approximately 4.5 × 10^5^) with cisplatin (CisPt) or leader hexapeptide conjugated with cisplatin (LP-CC6) or rMnSOD 24 leader peptide conjugated with cisplatin (LP-CC24) was evaluated by atomic absorbance spectrophotometry. All cell lines were incubated with 11.1 μg of Pt (92 μM) in all treatments (CisPt and LP-CC treatments). The results presented in Table 1 show the uptake of CisPt for the different treatments in cultured cell lines, demonstrating how the hexapeptide enters cells and that the peptide LP-CC6 delivered ~2 to 8 times more CisPt into the cells than CisPt alone. The significance of the differences between treatments found in this study was summarized according to the following parameter: the uptake of CisPt is expressed as the ratio (i.e., percentage) between the level of Pt in pellets compared with the supernatant. For each treatment and cell line, the statistical analysis was based on three independent replicates^20^. Based on the data in this table, we can conclude that LP-CC6 showed a significantly greater uptake than all other treatments in A2780 (5.91), MCF-7 (4.30), A375 (15.50), DU145 (46.97) and HuH-7 (38.11) cells; however, in PaTu-8902 cells, LP- CC24 demonstrated a greater uptake. The presence of Pt was also evaluated in ER^–^ MDA-MB-231 cell line. LPCC-6 showed a significantly lower uptake (Mean = 0.41; Var = 6.05 E-06; P- value = 1.0).

### Cytotoxicity assay

Synthetic LP-CC6, LP-CC24 and CisPt alone were added to the culture medium at a final concentration of 1 μM. Treated tumour cells were analysed for cell lysis by measuring Lactate Dehydrogenase (LDH) release. LDH release into the culture supernatant was expressed as a percent of total LDH (i.e., from cell lysis induced by detergent treatment). Approximately 3% LDH was found in the medium of untreated cultures.

Table 2 shows the cytotoxic activity exerted by LP-CC6 and illustrates the mean level estimates of LDH based on the subsequent triplicate treatments of cell lines.

LP-CC6 exhibited significantly higher activity, except in PaTu-8902 cells, with a noticeable peak in ovarian (85.0%), breast (73.0%) and hepatocellular carcinoma (69.0%) cells.

For each cell line, the table reports the estimate of the mean (sample mean), the unbiased estimate of the population variance (sum of squares divided by sample size minus 1), and the p-values to test the null (one-sided) hypothesis that the mean level of LDH for the treatment in a row is less than or equal to the mean level of LDH for the treatment in the column against the alternative that the former is greater than the latter. The estimates of the population variance revealed clear evidence of heteroscedasticity; the p-values were computed using the one-sided Welch’s test for the difference between means based on independent standard samples with unequal variances^20^. The approximate degree of freedom was rounded to the next lowest integer.

### Confocal analysis

**Figure 1:**
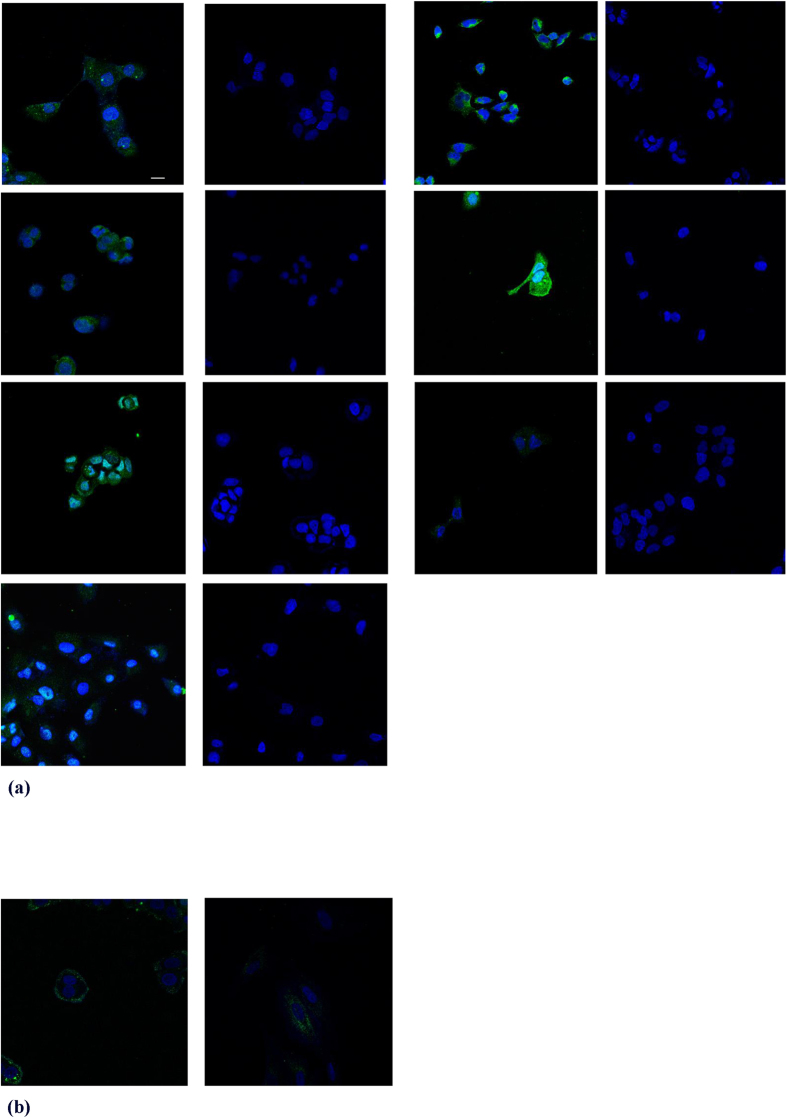
Representative confocal internal plane images of cell lines. (**a**) confocal images of the internalization of the hexapeptide in the cytoplasm. In the first column respectively PaTu-8902, A2780, MCF-7, HuH-7 and in the second column the control of each adjacent cell line; in the third column respectively A375, DU145, MCF-10A and in the fourth column the control of each adjacent cell line. Note the green fluorescence dispersed in the cytoplasm. No labelling of the controls was observed. This figure is a merged image of green and blue fluorescence images (exc. 488 nm Argon Laser/em. BP500–550 filter). Scale bar = 14 μm for all images except for A375 and its control = 25 μm. (**b**) confocal images of the internalization of the scrambled hexapeptide in the cytoplasm of MCF-7 and MCF-10A cell lines. Scrambled does not enter both cells. It is detectable outside the cell membrane. Controls are not shown.

Internalization of the hexapeptide in the cytoplasm of MCF-7, PaTu-8902, DU145, A375, A2780, HuH-7 and MCF-10A cells was demonstrated using confocal microscopy. Incubation with 92 μM hexapeptide-FITC for 3 hours revealed diffuse green fluorescence of the peptide, enhanced by the use of the antibody II Alexa 488A, by the indirect immunofluorescence method. Movement of the confocal image plane inside the cells demonstrated that the detected fluorescence is located in the cells. The diffuse green fluorescence of the peptide was mainly detectable in the inner part of the cytoplasm of all types of cancer cell lines used and also in the nuclei of A375 and HuH-7 cells and slightly in the nuclei of PaTu cells (Fig. 1a). Incubation with 92 μM scrambled hexapeptide-FITC for 3 hours revealed peripheral green fluorescence of the peptide demonstrating that the detected fluorescence is located outside the cytoplasmic membrane of MCF-7 and MCF-10A cell lines (Fig. 1b).

### Clonogenic assay

**Figure 2:**
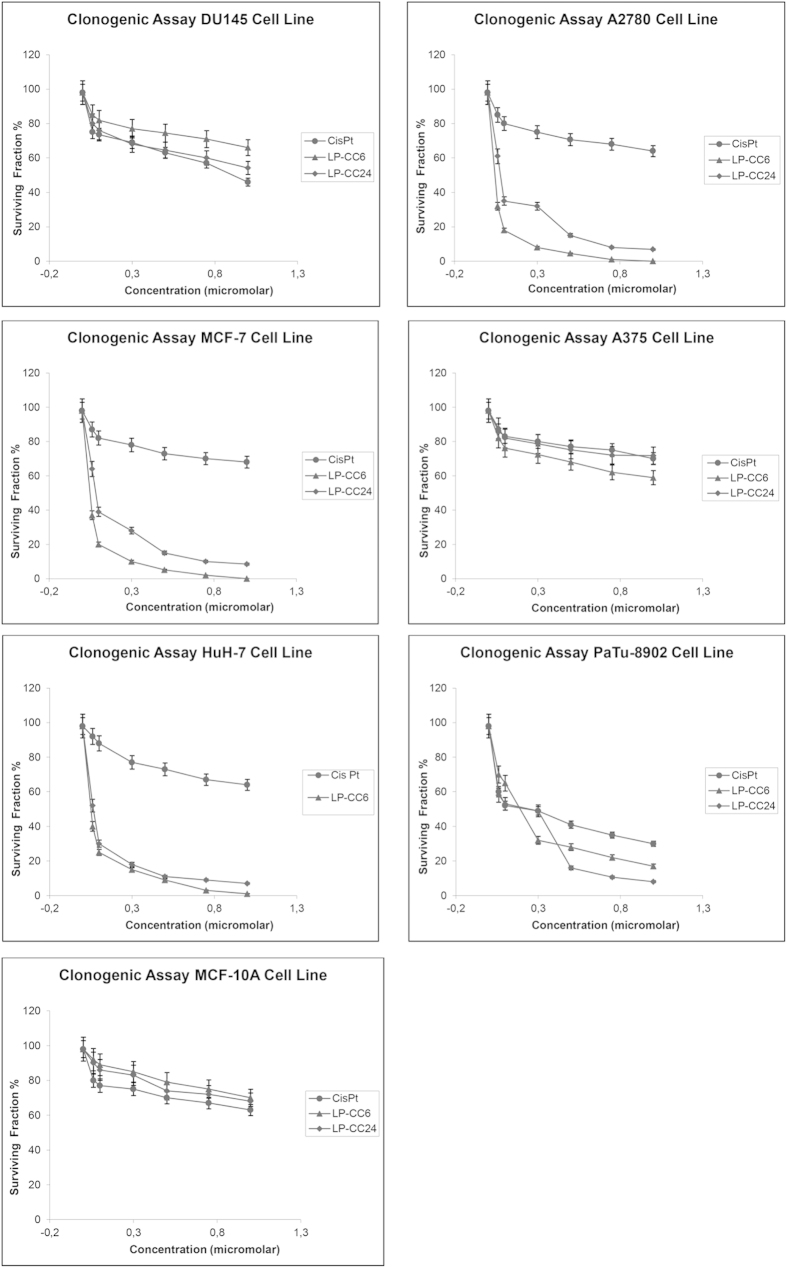
Clonogenic assay. Figure 2 shows the cell surviving fraction observed after drug treatment with LP-CC6 ranging from 0% (MCF-7, A2780 cell lines) to 58% (A375 cell line) and, for LP-CC24, from 7% (HuH-7) to 71.8% (A375) while the surviving fraction of tumour cells in the presence of CisPt alone at the same concentration was approximately 60%. The plating efficiency and the surviving fraction for each cell line after each treatment were calculated according to the method proposed by Franken *et al.*^35^ and averaged approximately 80% for all cell lines. Survival was calculated compared with non-treated samples using an average of three determinations of the same dose rate of cells (±SE). The average values with a standard deviation were determined from three independent experiments using different LP-CC6, LP-CC24 and CisPt preparations.

The clonogenic test using normal MCF-10A cells in the presence of CisPt alone exhibited a cell surviving fraction of 63% at the maximal dose of 1.0 μM. When the MCF-10A cells were treated with 1.0 μM LP-CC6, the cell surviving fraction was 70% (Fig. 2). In tumour cells (i.e., MCF-7, A2780, PaTu-8902, HuH-7, DU145 and A375), 1 μM LP-CC6 resulted in a cell surviving fraction ranging from 0% (MCF-7, A2780) to a maximum of 58% (A375); with regard to LP-CC24, the cell surviving fraction ranged from 7% (HuH-7) to 71.8% (A375), while the surviving factor of tumour cells in the presence of CisPt alone at the same concentration was almost always approximately 60% (Fig. 2).

### Real-time PCR analysis

The effects of LP-CC6, LP-CC24 and CisPt on tumour cells were investigated by real-time PCR (qPCR) by evaluating the expression of the *TP53* gene that encodes a tumour suppressor protein, the pro-apoptotic *BAX* gene and the *BCL2* gene, which encodes an integral outer mitochondrial membrane protein that blocks the apoptotic death of some cells, such as lymphocytes.

**Figure 3:**
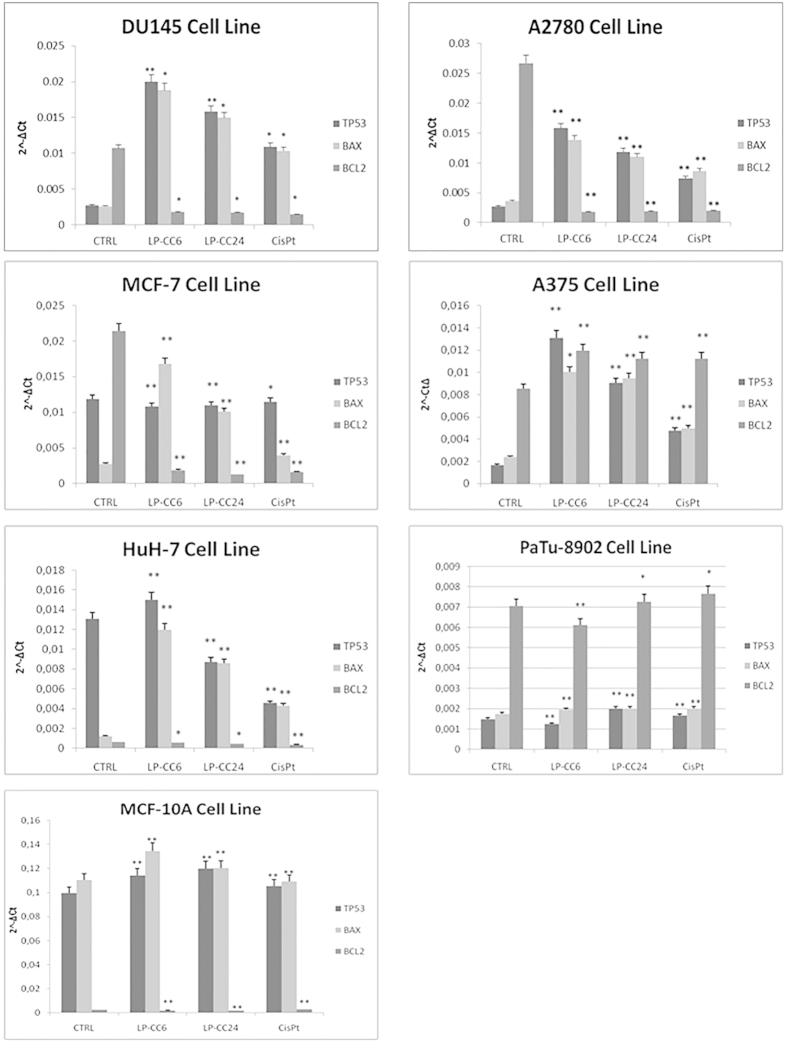
Real-time RT-PCR analysis. Figure 3 shows analysis of mRNA levels of *TP53, BAX, BCL2* in all cells treated with LP-CC6, LP-CC24 and CisPt alone by Real-time RT-PCR. We observed increased mRNA levels of *TP53* and *BAX* after 3 hours of treatment with LP-CC6. The expression of these genes was lower in the presence of LP-CC24 in all cell lines except for the melanoma (A375) and pancreatic carcinoma (PaTu- 8902) cell lines. In these cells, all three drugs exhibited little effect on the expression of the transcripts examined. Also evident is a downregulation of the transcript for *BCL2*, well represented in almost all control cell lines, after treatment with LP-CC6 and LP-CC24. Each sample was normalized to human *GAPDH*. The bars indicate the mean values of three independent experiments (the bars indicate the s.d.; **p-value < 0.005; *p-value < 0.05; Student’s t-test).

To verify the effect of treatment with LP-CC6, LP-CC24 and free CisPt, we analysed changes in the expression of the transcripts for *TP53, BAX,* and *BCL2* by real-time PCR. Figure 3 shows the real- time PCR output from cell lines treated for 3 hours with LP-CC6, LP-CC24 and CisPt alone at the same molar concentration containing 11.1 μg of the drug. We observed increased mRNA levels of *TP53* and *BAX* after 3 hours of treatment with LP-CC6. The expression of these genes was lower with LP-CC24 in all cell lines except in the melanoma cell line (A375) and in pancreatic carcinoma (PaTu-8902). In these cells, all three drugs exerted little effect on the expression of the transcripts examined. Also evident is a downregulation of the *BCL2* transcript, which was well represented in almost all control cell lines, after treatment with LP-CC6 and LP-CC24, confirming the results obtained with the LDH and clonogenic assays (Fig. 2).

**Figure 4:**
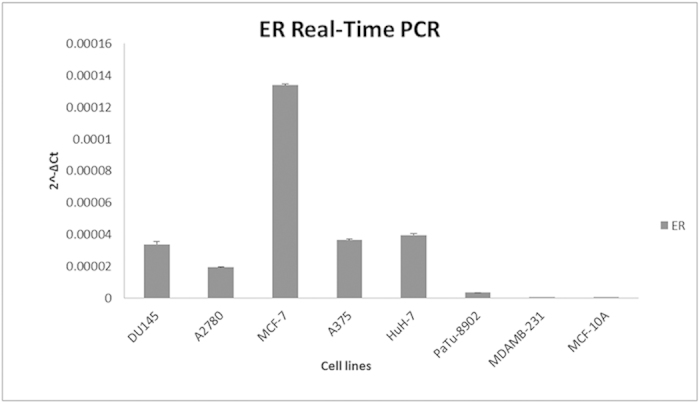
ER expression analysis by Real-time RT-PCR. Figure 4 shows analysis of mRNA levels of *ESR* in all cells examined by Real-time RT-PCR. We observed higher level of *ESR* in MCF-7 and a moderate and/or low expression in the other cell lines. Each sample was normalized to human *GAPDH*. The bars indicate the mean values of three independent experiments (the bars indicate the s.d.; **p-value < 0.005; *p-value < 0.05; Student’s t-test).

The expression of ER gene was evaluated by a qPCR experiment in all the cell lines examined. A high level of ER was observed in MCF-7 and a moderate and/or low expression in the other cell lines (Fig. 4).

## Discussion

We previously demonstrated the cytotoxic activity of rMnSOD^16^. We also demonstrated that the 24- aa leader peptide alone plays a role in rMnSOD’s ability to enter tumour cells expressing the ER and deliver either rMnSOD or other molecules bonded to it^18^.

We reacted the synthetic 24-aa leader peptide of rMnSOD with the ER to identify which amino acids were participating in the bond. Surprisingly, we found that only six of the 24 amino acids were involved in the reaction with a specific ER sequence (i.e., ***SIILLNSGVYTFLSSTLKSLEE**)*. These amino acids represent the gate through which the hexapeptide with the whole rMnSOD can enter the cells. This knowledge allows us to assign the role of molecular carrier to this hexapeptide and propose its therapeutic use for cells expressing the ER sequence.

The valuable properties of this molecular carrier include its ease of handling, reduced antigenicity, low cost, and relative ease of production. These characteristics may provide an opportunity to formulate new drugs with specific and selective effects for use in therapy. In fact, molecules showing therapeutic or diagnostic activities (such as radioactive molecules for imaging analysis) could be conjugated to this hexapeptide and injected. In this way, targeted treatment could be delivered inside diseased ER^+^ cells. The correct MW was confirmed by mass spectrometric (MALDI-TOF) analysis. In all performed experiments, no toxic alterations were observed in cells displaying internalization of the hexapeptide alone. Confocal microscopic analysis of all focal planes revealed that the FITC-peptide penetrated cancer cells by entering the cytoplasm, where it was detected in all treated cells. These results are in agreement with previous immunocytochemical detection reports using a polyclonal antibody directed against the 24-aa leader sequence of rMnSOD^21^. To verify the capacity of the hexapeptide to deliver molecules into tumour cells, a synthetic construct, LP-CC6, was prepared. Quantitative CisPt determination demonstrated that LP-CC6 delivered high amounts of platinum into the cells, more than that released into cells when CisPt was used alone.

The atomic absorbance spectrophotometric analysis required, in the experiments with LP-CC6, a high concentration of CisPt conjugated to the hexapeptide (i.e., 11.1 μg, corresponding to 92 μM). However, the toxic effect of LP-CC6 is already high at a concentration of 1 μM for 48 hr (i.e., LDH release, clonogenic assay). This is significantly lower than the concentration required to CisPt alone to induce apoptosis in MCF-7 cells (i.e., 80–160 μM for 24 h)^22^. It is important to note that, despite a positive uptake of LP-CC6 by normal cells, apoptosis was undetectable, as confirmed by LDH release (Table 2) and *BAX, TP53,* and *BCL2* gene expression (Fig. 3).

The reason for differences in uptake may be related to the fact that the low amount of CisPt contained in LP-CC6 may not be sufficient to reach the toxic threshold, given that, in normal cells, there are detectable levels of antioxidant enzymes (i.e., MnSOD and catalase). In normal cells, these enzymes can neutralize the cytotoxic effect of CisPt-derived free radicals. In contrast, the amount of CisPt taken up by cancer cells is highly toxic because these enzymes, particularly catalase, are poorly expressed in cancer cells^12^.

The cytotoxic activity exerted by the hexapeptide conjugated to CisPt was significantly high, with a particular peak of activity in ovarian, breast, prostatic and hepatocellular carcinoma cells. Less cytotoxicity was exerted on pancreatic cells by LP-CC6, but it was still sufficient to kill cancer cells. We are unable to explain the reasons for the variations in cytotoxicity across cell lines; however, some hypotheses can be formulated regarding this effect. The most likely explanation is that there is a different level of expression of oestrogen receptors in these cells (Fig. 4). The analysis of expression of ER gene by a qPCR experiment confirmed that the increased cytotoxicity is related to the different expression of ER in cells. Several authors investigated the correlation between cisplatin cytotoxicity and ER presence. Some described that the treatment of human cancer cells that have steroid hormone receptors with the appropriate hormone, oestrogen and/or progesterone, significantly increased the potency of cisplatin by causing the overexpression of HMG1. These endogenous HMG1 proteins were already involved in complexes with chromatin and transcription factors. They, expressed as a scaffold to facilitate ER- or PR-mediated transcription, may be more readily available to bind to cisplatin-DNA intra-strand crosslinks^23^. Other findings suggested that HMG1 induction as an enhancer of platinum sensitivity was mediated through interaction between oestrogen and ER-α in patients with prostate cancer^24^. Another evidence of the correlation between cisplatin cytotoxicity and ER presence was that several oestrogen-tethered platinum (IV) complexes (BEP) were prepared. They were inspired by the observation that oestrogen receptor-positive cells exposed to the hormone are sensitized to cisplatin^25^. Moreover, in MCF7 cells in which oestrogen receptor is activated, DNA damage inhibited checkpoint cell cycle, which resulted in less effective DNA repair. In the cancer cells, where oestrogen receptor is inhibited, there was a better DNA repair and improved cell survival, which decreased the cytotoxic action of cisplatin^26^. BRCA1 has been proved to interact physically with ER-α and inhibit its transcriptional activity. BRCA1-defective cells displayed significantly higher sensitivity to cisplatin supporting the idea that cisplatin-induced DNA damage is strongly associated with the inactivation of BRCA1^27^.

We are preparing a specific antibody against the aa sequence of the ER to verify the correlation between ER and LP. This sequence could crosslink with the leader peptide when entering the cells. CisPt is a drug widely used in chemotherapy that causes damage to DNA by alkylation and determination of crosslinks. The exact processes responsible for CisPt-induced cell death and the processes involved in regulating CisPt sensitivity are still being studied^22^. One of the major problems caused in the clinic by use of CisPt is the appearance of severe dose-dependent side effects. Because of these side effects, many tumours become resistant due to high dosage or long- term treatment. Moreover, it is important to highlight that, to achieve significant toxicity, as in the case of MCF-7 cells (but also equally significant in other tumour cells) when using CisPt alone, it was necessary to use at least a 25 μM concentration, corresponding to 730 ng of CisPt. However, when using LP-CC6, to produce the same cytotoxicity, only a 1.0 μM concentration was needed, corresponding to only 30 ng of CisPt.

We chose to use real-time PCR to evaluate the expression of three genes (*TP53, BAX, BCL2*) that are involved in apoptosis. Our data^18^ showed that both the 24-aa leader peptide linked to CisPt, as well as the hexapeptide linked to CisPt, at the low concentrations used, activate apoptotic genes (i.e., *BAX* and *TP53*) while inhibiting the antiapoptotic gene *BCL2*, in line with reports in the literature^28^,^29^,^30^,^31^,^32^,^33^,^34^.

It is hence easy to note that the delivery of a small but critical amount of CisPt carried by LP-CC6 and released directly into tumour cells resulted in an increased therapeutic index of CisPt, which produced more marked cytotoxicity in tumour cells and increased tumour cell death. On the other hand, it is well known that the therapeutic effect of drugs for tumour cells is related to the lesion being exposed to a critical (and not necessarily plasmatic) concentration of the molecule as well as to a high retention of the drug or its metabolites^35^,^36^,^37^,^38^. These are exactly the conditions generated by the LP-CC6 injection, which led to an increased therapeutic index of CisPt.

The data we obtained were significant, and the main results from the cytotoxicity data can be summarized as follows: LP-CC6 displayed a greater cytotoxic effect (i.e., was more effective) than CisPt in all cancer cell lines with the exception of PaTu-8902 cells and A375 cells, in which CisPt alone or linked to peptides was most effective. Many authors have studied the mechanisms of cellular resistance to apoptosis induced by chemotherapy, particularly in pancreatic cancer cells^39^. Interestingly, in normal MCF-10 cells, LP-CC6 was less toxic than usual. A key ability of a drug is to penetrate target cells, and the hexapeptide clearly demonstrated this skill. We found that the hexapeptide delivered and released 2 to 8 times more CisPt than the 24-aa leader peptide in all tumour cells except for PaTu-8902, MCF-10A and MDA-MB-231 cell lines. This observation demonstrates that CisPt fails to reach the site of tumour lesions, and this may be the case for other cytostatics as well. This observation is not of secondary importance; in fact, because cytostatic agents are antireplicative, mutagenic and oncogenic, they should be used in patients with caution in very small doses to minimize or eliminate side effects due to high toxicity. Unfortunately, their inability to reach and penetrate tumour lesions^18^ leads clinicians to use high doses of these drugs, which results in unpleasant side effects for the patients. Therefore, the use of the hexapeptide as a molecular carrier could help ameliorate the therapeutic effects by increasing the internalized dose, which would reduce the given dose and thereby minimize the side effects.

In conclusion, the present study provides strong evidence that the hexapeptide, when conjugated to CisPt and added to cells *in vitro*, penetrated cells through an aa sequence of the ER and delivered high amounts of CisPt. The aa sequence of the ER represents a gate through which the rMnSOD or any other molecule bound to a hexapeptide can be delivered into the cells. This targeted delivery increases the therapeutic index of CisPt, which, despite its low dose, was able to kill cancer cells. Finally, because the hexapeptide avoids biological barriers, quickly enters cells and is easy to administer, we believe that this construct is more versatile than the original 24-aa peptide and should, therefore, be considered as an anticancer agent. We conclude that cell penetration is an essential prerequisite for signal transduction therapy, therapies influencing transcription and translation, and thus, effective tumour therapy; additionally, it may be useful for gene therapy in the future.

## Materials and Methods

### Reagents

The 24-aa rMnSOD leader peptide, the new leader hexapeptide (LP; ***AVCGTG***) and the scrambled hexapeptide (***GAVCGT***) were obtained according to described methods. The anti- 24-aa leader peptide primary polyclonal antibody (rabbit) was distributed by PRIMM BIOTECH (Milan, Italy). This antibody was used at a concentration of 1:200. CisPt was purchased from Bristol-Mayer Co. (New York, NY, USA).

### Synthesis of hexapeptide (*AVCGTG*) and of scrambled hexapeptide (*GAVCGT*) conjugated to fluorescein

The peptides were synthesized step-wise in batch-mode on a solid support with an automatic synthesizer, Syro (MultiSynTech), using Fmoc/tBu chemistry^40^ starting from the C- terminal residue pre-loaded on PS-PHB resin with an average of 0.57 mmol/g substitution (Rapp Polymere). Synthesis on a 30 micromol scale proceeded through standard cycles of Fmoc de- protection [piperidine: DMF, 0.2:1(v/v)] and Fmoc- amino acid coupling [Resin: Fmoc-amino acid: HBTU: DIEA, 1:4:4:8]^41^.

**Amino acid derivatives for hexapeptide (*****AVCGTG***): Fmoc-Ala-OH, Fmoc-Val-OH, Fmoc- Cys (Trt)-OH, Fmoc-Gly-OH, Fmoc-Thr (tBu)-OH.

**Amino acid derivatives for scrambled hexapeptide (*****GAVCGT***): Fmoc-Ala-OH, Fmoc-Cys (Trt)-OH, Fmoc-Gly-OH, Fmoc-Thr (tBu)-OH, Fmoc-Val-OH. Fluorescein was coupled to a peptidil-resin using 5(6)-FAM:HBTU:DIEA in a 4:4:8 ratio.

### Cleavage

Side chain de-protection with a concomitant cleavage of the peptides from a solid support were obtained by suspending the protected peptide resin in a mixture of TFA:water [95:5 (v/v)] for three hours. The resin was removed by filtration under reduced pressure. Precipitation was achieved by collecting the filtrate on a bed of cold ether. After several ether washes, the crude compounds were suspended in water (0.1% TFA, v/v) and lyophilized.

### Analysis

Purity was assessed by analytical RP-HPLC (Shimadzu) using a Vydac C18 column (4.6 × 150 mm) with eluent A (0.1%TFA in water) and eluent B (0.1%TFA in acetonitrile) over a gradient spanning 5% B to 65% B over the course of 20 min at a flow rate of 1 mL/min (R.t.) for 16.5 min. Identity was assessed by mass spectrometry performed on a MALDI-TOF spectrometer (Applied BioSystem) [MH+ = 866].

### Synthesis of the hexapeptide (*AVCGTG*) conjugated to cisplatin

The peptide was synthesized step-wise in batch-mode on a solid support with an automatic synthesizer, Syro (MultiSynTech), using Fmoc/tBu chemistry^40^ starting from the C-terminal residue pre-loaded on PS-PHB resin with an average of 0.57 mmol/g substitution (Rapp Polymere). Synthesis on a 30-micromol scale proceeded through standard cycles of Fmoc deprotection [piperidine: DMF, 0.2:1(v/v)] and Fmoc-amino acid coupling steps [Resin: Fmoc-amino acid: HBTU: DIEA, 1:4:4:8]^41^. As a Pt binder, diamino-ethyl-glycine was used, which, by virtue of the presence of two free amine groups, can combine platinum (II) ions as PtCl2^19^.

**Amino acid derivatives**: Fmoc-Ala-OH, Fmoc-Val-OH, Fmoc-Cys (Trt)-OH, Fmoc-Gly-OH, Fmoc-Thr (tBu)-OH.

**Binder**: N-Fmoc [N′-Fmoc-(2′aminoethyl)] glycine.

### Cleavage

Side chain de-protection with concomitant cleavage of the peptide from a solid support was achieved by suspending the protected peptide-resin in a mixture TFA and water [95:5 (v/v)] for three hours. The resin was removed by filtration under reduced pressure. Precipitation was achieved by collecting the filtrate on a bed of cold ether. After several ether washes, the crude compound was suspended in water (0.1% TFA, v/v) and lyophilized.

### Analysis

Purity was assessed by analytical RP-HPLC (Shimadzu) using a Vydac C18 column (4.6 × 150 mm) with eluent A (0.1% TFA in water) and eluent B (0.1% TFA in acetonitrile) over a gradient spanning 5% B to 65% B over the course of 20 min at a flow rate of 1 mL/min. (R.t.) for 16.5 min. Identity was assessed by mass spectrometry performed on a MALDI-TOF spectrometer (Applied BioSystem) [MH+ = 870].

### Purification

RP-HPLC purification was performed on a Vydac C18 column (22 × 250 mm) with eluent A (0.1% TFA in water) and eluent B (0.1% TFA in acetonitrile) at a gradient from 5% B to 65% B over the course of 40 min at a flow rate of 20 mL/min).

### Cell lines

The cell lines used in this study included MCF-7 human mammary cancer cells (ATCC, HTB-22), A2780 human ovarian cancer cells (Sigma, 93112519), PaTu-8902 pancreatic adenocarcinoma cells (DSMZ, n° ACC179), HuH-7 human hepatocarcinoma cells (JCRB Cell Bank JCRB043, National Institute of Biomedical Innovation), MCF-10A normal breast cell (ATCC, CRL-10317), DU145 prostatic adenocarcinoma (ATCC, HTB-81), A375 human melanoma cells (ATCC, CRL 1619) and MDA-MB-231 human mammary cancer cells (ATCC, HTB-26). All cells were grown in Dulbecco’s modified Eagle’s medium supplemented with 10% foetal bovine serum. The cells were incubated at 37 °C in a humidified atmosphere with 5% CO_2_ .

### Uptake of cisplatin into tumour cells evaluated by atomic absorbance spectrophotometric analysis

Samples of each target cell used in the present study were treated for 2 hours in the presence of 92 μM CisPt, 92 μM LP-CC24 and 92 μM LP-CC6 cultured in DMEM with 10% FCS (Gibco, Life Technologies Italia). The controls were obtained by maintaining the same cells under the same culture conditions but with the presence of 92 μM of the 24-aa leader peptide alone or 92 μM of hexapeptide, both in the absence of CisPt. Given the sensitivity of this method, the amount of the sample must also be considered. It must be highlighted that for the determination of platinum using a spectrofluorimeter, it was necessary to use a high concentration of platinum conjugated to the hexapeptide, i.e., 11.1 μg or a 92 μM concentration in molar terms. Determinations are performed in triplicate. Target cells were collected, washed twice with phosphate buffer saline 1X (PBS) and treated with 50 μL of 35% HNO_3_ for 16 hours. After a 3-hour incubation, Pt content in the growth medium and the cell pellet was determined by graphite furnace atomic absorption spectrophotometry (Analyst 800, Perkin–Elmer, Norwalk, CT, USA) using the following parameters: pre-treatment temperature, 1,300 °C; atomization temperature, 2,200 °C; and matrix modifer, 0.015 mg of Pd (Palladium) and 0.01 mg of Mg(NO_3_)_2_. Measurements were performed using a graphite furnace equipped with a Zeeman-effect background correction system. A pyrolytic graphite-coated THGA tube (Perkin–Elmer) with an integrated Lvov-type platform was used for metal determination. A Pt standard in 2.5% HNO_3_ (Spectrascan) was used as a stock solution for the construction of the 3-point calibration curve. Each measurement was performed in triplicate.

### Cytotoxicity assay

Cell lysis induced by LP-CC6 treatment was measured by the release of the lactate dehydrogenase (LDH) enzyme. LP-CC24, LP alone or CisPt alone were added to exponentially growing cultures of MCF-7, A2780, PaTu-8902, HuH-7, MCF-10A, DU145 and A375 cells at a final concentration of 1 μM. The concentration of 1 μM was chosen because we previously demonstrated that, while treating MCF-7 and other tumour cells with increasing concentrations of LP-CC6 (0.06 to 36.8 μM) for 48 hours, the optimal concentration of LP-CC6 based on a LDH assay was between 0.5 and 1 μM^18^. LDH released into the culture supernatant was expressed as the percent of total LDH from cell lysis induced by detergent treatment. Approximately 3% LDH was found in the medium of untreated cultures. LDH release into the medium supernatant was measured at the indicated times post-application (i.e., 48 hours) using the CytoTox 96 nonradioactive cytotoxicity assay kit from Promega (Promega Italia, S.r.l. Milan, Italy) according to the manufacturer’s recommendations. Experiments were performed in triplicate using different preparations.

### Confocal analysis

MCF-7, PaTu-8902, DU145, A375, A2780, and HuH-7 cell lines underwent direct and indirect immunofluorescent assays for hexapeptide detection. In order to detect the scrambled hexapeptide direct and indirect immunofluorescent assays were performed on MCF-7 and MCF-10A. For both methods of detection, all cells were incubated with 92 μM AVCGTG-FITC for 3 hours in glass-bottom dishes (MatTek Corporation, Ashland, MA, USA), fixed with 4% paraformaldehyde/PBS for 15 min and rinsed twice with 1X PBS containing 0.1% Triton to remove traces of fixative. For indirect immunofluorescence, after washes and permeabilization with 0.4% Triton X-100/PBS for 15 min, blocking was performed with normal horse serum NGS (Vector, USA), 3% BSA, and 0.1% Triton X-100/PBS. Cells were incubated with the anti-24-aa leader peptide primary polyclonal antibody (rabbit) overnight at 4 °C. After three washes with 1X PBS, cells were incubated with CF 488 A (Biotium, CA) for 2 hours in the dark at room temperature. Untreated cell samples (i.e., without AVCGTG-FITC) were used for the background control. For both immunofluorescence methods, nuclei were stained with 4′, 6-diamidino-2-phenylindole (DAPI; 10 μg/mL). The slides were stored in the dark at 4 °C and then observed on a confocal microscope (LSM 510 META, Zeiss).

### Clonogenic tests on normal and tumour cells following LP-CC6, LP-CC24, and CisPt treatment

Confluent 75-cm^2^ flasks of tumour (MCF-7, A2780, HuH-7, DU145, A375, PaTu-8902) or normal (MCF-10A) cells were trypsinized, counted with a haemocytometer and diluted with incomplete media to obtain 100 cells ⁄ mL. Two millilitres of cell suspension was plated in each well (BD Falcon six-well tissue culture plate) to obtain 200 cells per well. Normal and tumour cells were treated in the presence of LP-CC6, LP-CC24 and CisPt at final concentrations of l μM. Experiments were performed in triplicate. Colonies were stained with crystal violet after 14 days, and those colonies containing at least 30 cells were counted as surviving colonies. The plating efficiency and the survival fraction for each cell line after each treatment were calculated according to the method proposed by Franken *et al.*^42^ and averaged approximately 80% for all cell lines. Survival was calculated in comparison with non-treated samples, using an average of three determinations of the same dose rate of cells (±SE). Average values with a standard deviation were determined from three independent experiments using different LP-CC6, LP-CC24 and CisPt preparations.

### Real-time PCR assay in cells treated with LP-CC6, LP-CC24 and CisPt

Target cells: three independent samples of each of the target cells used in the present study (MCF-7, MCF-10A, A2780, A375, HuH-7, DU145 and PaTu-8902) were treated for 3 hours with 92 μM of CisPt or 92 μM LP-CC6 (containing an equivalent CisPt concentration) cultured in DMEM with 10% FCS (Gibco). The controls were obtained by maintaining the same cells in identical culture conditions but in the presence of 92 μM of peptide alone in the absence of CisPt. RNA extraction: the RNeasy Plus Mini Kit (Qiagen, Hilden, Germany) was used to extract total RNA from target cells. Contaminating genomic DNA was removed using gDNA Eliminator spin columns (Qiagen). The purity and integrity of the isolated RNA were determined using a spectrophotometer, denaturing agarose gel electrophoresis and an Agilent 2100 Bioanalyzer (Agilent, Santa Clara, CA, USA). The RNA integrity values of the isolated RNA were nearly 10. Total RNA, isolated from cells as described above, was used for cDNA synthesis. RT-PCR analyses of the tumour protein p53 gene (*TP53*), Bcl-2-associated X protein gene (*BAX*) and B-cell CLL/lymphoma 2 gene (*BCL2*) were performed with ABI PRISM 7000 Sequence Detection System instruments and software (Applied Biosystems, Foster City, CA, USA) using the Power SYBR Green PCR Master Mix (Applied Biosystems) with the thermocycler conditions recommended by the manufacturer. RT-PCR analyses of oestrogen receptor gene (*ESR*) were also performed. All reactions were performed in a total volume of 25 μL containing 50 ng of cDNA and 2.5 μL of 10X QuantiTect Primer (Qiagen). Reactions were performed in triplicate on three independent sets of RNA. The QuantiTect Primers have been bioinformatically validated to detect RNA only, provided that no pseudogenes with high cDNA similarity exist and the transcript is not derived from a single-exon gene. Negative controls (i.e., no RNA added) were processed under the same conditions as experimental samples. Human glyceraldehyde 3-phosphate dehydrogenase (*GAPDH*) was used as an endogenous control to normalize target gene expression (ΔCt) and to correct for experimental variation^43^. The relative gene expression was calculated using the method described by Livak and Schmittgen^44^ and is expressed as the fold change compared with the control.

## Statistical Analysis

All data related to real-time PCR are shown as the mean ± s.d. Student’s t-test was used to determine the significance of the observed data.

## Additional Information

**How to cite this article**: Borrelli, A. *et al.* A new hexapeptide from the leader peptide of rMnSOD enters cells through the oestrogen receptor to deliver therapeutic molecules. *Sci. Rep.* **6**, 18691; doi: 10.1038/srep18691 (2016).

## Supplementary Material

Supplementary Information

## Figures and Tables

**Table 1:** Uptake of cisplatin for the different treatments in cultured cell lines.

CELL LINE	TREATMENTS	Uptake Estimates		P-values (***)		
		Mean (*)	Var (**)	LP-CC6	LP-CC24	CisPt
DU145	LP-CC6	46.97	8.00E-02	0.00	–	0.00
	LP-CC24	13.84	4.72E-06	–	1.00	0.00
	CisPt	0.91	8.52E-08	1.00	1.00	–
A2780	LP-CC6	5.91	1.19E-04	–	0.00	0.00
	LP-CC24	0.65	5.22E-05	1.00	–	1.00
	CisPt	0.75	5.35E-06	1.00	0.00	–
MCF-7	LP-CC6	4.30	4.13E-05	–	0.00	0.00
	LP-CC24	2.60	5.49E-06	1.00	0.00	–
	CisPt	0.41	6.05E-06	1.00	–	1.00
A375	LP-CC6	15.50	8.89E-05	–	0.00	0.00
	LP-CC24	4.45	1.15E-03	1.00	–	0.00
	CisPt	1.81	4.30E-08	1.00	1.00	–
HuH-7	LP-CC6	38.11	7.29E-06	0.00	–	0.00
	LP-CC24	5.26	2.15E-06	–	1.00	0.00
	CisPt	1.29	1.86E-06	1.00	1.00	–
PaTu 8902	LP-CC6	2.83	6.82E-07	–	1.00	0.00
	LP-CC24	17.00	1.08E-05	0.00	–	0.00
	CisPt	2.45	1.13E-08	1.00	1.00	–
MCF-10A	LP-CC6	4.29	8.48E-06	–	0.98	0.00
	LP-CC24	4.34	2.21E-04	0.02	–	0.00
	CisPt	1.69	6.95E-06	1.00	1.00	–

In Table 1, LP-CC6 showed significantly greater uptake than all other treatments in A2780 (5.91), MCF-7 (4.30), A375 (15.50), DU145 (46.97) and HuH-7 (38.11) cells; in PaTu-8902

cells, LP-CC24 demonstrated a greater uptake. Uptake of CisPt for the different treatments in cultured cell lines, showing how the hexapeptide enters cells. The data were obtained from 3 independent observations (the bars indicate the s.d.). The mean level estimates of the uptake of CisPt in cell lines treated with LP-CC6, LP-CC24 and CisPt; (N = 3).

*Sample mean and **unbiased estimate of the population variance. ***based on Welch’s test^20^ for the null hypothesis that the mean level of uptake for the treatment in the row is less than or equal to the mean level of uptake for the treatment in the column, against the alternative that the former is greater than the latter.

**Table 2:** Cytotoxicity in cell culture lines induced by different treatments.

CELL LINE	TREATMENTS	LDH Estimates		P-values (***)			
		Mean (*)	Var (**)	LP	LP-CC6	LP-CC24	CisPt
DU145	LP	18.2	0.367	1.000	1.000	–	1.000
	LP-CC6	54.2	0.108	0.000	–	0.000	0.000
	LP-CC24	36.0	0.369	–	1.000	0.000	0.003
	CisPt	32.0	0.056	0.997	1.000	0.000	–
A2780	LP	4.0	0.095	–	1.000	1.000	1.000
	LP-CC6	85.0	0.067	0.000	–	0.000	0.000
	LP-CC24	54.0	0.032	0.000	1.000	–	0.000
	CisPt	34.0	0.805	0.000	1.000	1.000	–
MCF-7	LP	22.0	0.036	–	1.000	1.000	0.990
	LP-CC6	73.0	0.042	0.000	0.002	–	0.000
	LP-CC24	68.0	0.334	0.000	–	0.998	0.000
	CisPt	25.0	0.771	0.010	1.000	1.000	–
A375	LP	4.5	0.032	–	1.000	1.000	1.000
	LP-CC6	38.0	0.411	0.000	–	0.002	0.004
	LP-CC24	33.6	0.005	0.000	0.998	–	0.972
	CisPt	35.0	0.543	0.000	0.996	0.028	–
HuH-7	LP	2.0	0.705	–	1.000	0.999	1.000
	LP-CC6	69.0	0.060	0.000	–	0.000	0.000
	LP-CC24	32.0	0.532	0.000	1.000	0.000	–
	CisPt	10.0	0.155	0.001	1.000	–	1.000
PaTu- 8902	LP	6.5	0.008	–	1.000	1.000	1.000
	LP-CC6	34.0	0.512	0.000	–	1.000	1.000
	LP-CC24	46.0	0.478	0.000	0.000	–	0.996
	CisPt	49.0	0.538	0.000	0.000	0.004	–
MCF-10A	LP	22.0	0.402	–	0.992	0.999	1.000
	LP-CC6	24.0	0.307	0.008	–	0.995	1.000
	LP-CC24	26.0	0.245	0.001	0.005	–	0.999
	CisPt	30.0	0.219	0.000	0.000	0.001	–

Table 2 shows the cytotoxic activity exerted by synthetic LP-CC6, LP-CC24 and CisPt alone and illustrates the mean level estimates of LDH based on the following triplicate treatments of cell lines. The hexapeptide showed more significant activity, except in PaTu-8902 cells, with a noticeable peak in ovarian (85.0%), breast (73.0%) and hepatocellular carcinoma cells (69.0%).

The data were obtained from 3 independent observations (the bars indicate the s.d.). The mean level estimates of LDH (cytotoxic effect) in the cell lines treated with LP, LP-CC6, LP-CC24 and CisPt;(N = 3).

*Sample mean and **unbiased estimate of the population variance. ***based on Welch’s test for the null hypothesis that the mean level of uptake for the treatment in the row is less or equal to the mean level of uptake for the treatment in the column, against the alternative that the former is greater than the latter.
