# Supplementary material for: A new hexapeptide from the leader peptide of rMnSOD enters cells through the oestrogen receptor to deliver therapeutic molecules
Source: Sci Rep. 2016 Jan 4;6:18691. doi: 10.1038/srep18691 (PMC4698655; doi:10.1038/srep18691)
Supplement: Supplementary Information [file srep18691-s1.pdf]

A new hexapeptide from the leader peptide of rMnSOD enters cells through the oestrogen receptor to deliver therapeutic molecules

Antonella Borrelli[1], Antonietta Schiattarella[9], Roberto Mancini[6], Alessandra Pica[2], Maria Laura Pollio[2], Maria Grazia Ruggiero[2], Patrizia Bonelli[1], Viviana De Luca[3], Franca Maria Tuccillo[1], Clemente Capasso[3], Enrico Gori[4], Marina Sanseverino[5], Andrea Carpentieri[7], Leila Birolo[7], Piero Pucci[7], Jean Rommelaere[8] and Aldo Mancini[9,*]

[1] *Molecular Biology and Viral Oncology, National Cancer Institute "Fondazione Pascale", Naples, Italy.*
[2] *Department of Biology, University of Naples, Federico II, Italy*
[3] *Institute of Protein Biochemistry, C.N.R., Naples, Italy*
[4] *Department of Statistics, University of Udine, Udine, Italy*
[5] *INBIOS - Institute of Genetic and Biophysics, C.N.R., Naples, Italy*
[6] *Department of Biotechnology, Friedrich-Loeffler-Institut, Neustadt, Germany*
[7] *Department of Chemical Sciences, University of Naples, Federico II, Italy*
[8] *Deutsches Krebsforschungszentrum, Infection and Cancer Program, Abt.F010, Heidelberg, Germany*
[9] *Laedhexa Biotechnologies Inc., QB3@953, San Francisco, CA, USA*

Mass Spectrometry Data

Crosslinking reaction. The crosslinking reaction between the 24-aa rMnSOD leader peptide and the ER was performed in 20 mM HEPES buffer using SULFO-EMCS (Pierce Biotechnology Inc. Rockford, IL, USA) as a bifunctional reagent. The ER (750 pmol) was divided into two aliquots, and 1.68 nmol of the 24-aa peptide was added to each aliquot. The sample aliquot (A) was then treated with 16.9 nmol SULFO-EMCS, whereas the second aliquot (B) was left untreated and used as a control. The reaction was performed for 16 hours at 4°C.

Reduction, alkylation, and enzymatic hydrolysis. Aliquots A and B were resuspended in 200 μL of denaturation buffer (guanidine 6 M, Tris 0.3 M, EDTA 10 mM, pH 8) and reduced using a 10:1 molar ratio of DTT:cysteines. After incubation at 37°C for two hours, carboxyamidomethylation was performed by 5:1 molar excess of iodoacetamide with respect to the thiol groups. The mixture was then incubated at room temperature for 30 min in the dark. The alkylation reaction was halted by lowering the pH with formic acid. Excess salts and reagents were removed by gel filtration on PD-10 columns. Elution was performed using 10 mM AMBIC buffer. Fractions were collected and analysed by a Beckman DU 7500 spectrophotometer, measuring the absorbance at 220 and 280 nm. Protein-containing fractions were collected and concentrated in a Speed-Vac centrifuge. Trypsin digestion was performed in 10 mM AMBIC buffer at 37°C for 12-16 hours using a 50:1 trypsin: protein ratio (w/w)[1].

MALDI-TOF/MS analysis. MALDI MS analyses were performed on a Voyager DE STR Pro instrument operating in reflectron mode (Applied Biosystems, Framingham, MA, USA). MALDI matrices were prepared by dissolving 10 mg of α-cyano in 1 mL of acetonitrile / 0.2% trifluoroacetic acid (70:30 v/v). Then, 1 µL of matrix was applied to the metallic sample plate, and 1 µL of the peptide mixture was added. Acceleration and reflector voltages were set up as follows: target voltage at 20 kV, the first grid at 66% of target voltage, delayed extraction at 200 ns.

References

1 Leo, G. *et al.* Proteomic strategies for the identification of proteinaceous binders in paintings. *Anal Bioanal Chem* **395**, 2269-2280, doi:10.1007/s00216-009-3185-y (2009).
